# Protocol Biopsies Reveal Progressive Arteriolar Thickening as a Predictor of Mortality in Kidney Transplant Recipients

**DOI:** 10.3390/life15101635

**Published:** 2025-10-20

**Authors:** Diana Rodríguez-Espinosa, Evelyn Hermida, Agustín Leal-Cúpich, Adriana García, Ana Belén Larque, Elena Cuadrado-Payán, Elena Guillén-Olmos, Marina Moncada, Pedro Ventura-Aguiar, David Cucchiari, Nuria Esforzado, Ignacio Revuelta, Fritz Diekmann, José Vicente Torregrosa, José Jesús Broseta

**Affiliations:** 1 Nephrology and Renal Transplantation, Hospital Clínic Barcelona, 08036 Barcelona, Spain; dmrodriguez@clinic.cat (D.R.-E.); hermida@clinic.cat (E.H.); agustin_leal88@hotmail.com (A.L.-C.); ecuadrado@clinic.cat (E.C.-P.); eguillen@clinic.cat (E.G.-O.); pventura@clinic.cat (P.V.-A.); cucchiari@clinic.cat (D.C.); nesforza@clinic.cat (N.E.); irevuelt@clinic.cat (I.R.); fdiekman@clinic.cat (F.D.); vtorre@clinic.cat (J.V.T.); 2 Department of Pathology, Hospital Clínic Barcelona, 08036 Barcelona, Spain; apgarcia@clinic.cat (A.G.); ablarque@clinic.cat (A.B.L.); 3 Facultat de Medicina i Ciències de la Salut, Universitat de Barcelona, 08036 Barcelona, Spain

**Keywords:** kidney transplantation, kidney graft biopsy, histopathology, cardiovascular events, all-cause mortality

## Abstract

Kidney transplant recipients remain at high risk of cardiovascular events and premature death. Whether chronic histological changes in protocol allograft biopsies provide prognostic information for patient outcomes beyond graft survival remains uncertain. In this prospective study of 458 kidney transplant recipients with biopsies performed at 3 and 12 months and followed up to 8 years, we assessed the association between vascular and interstitial lesions and major adverse cardiovascular events (MACEs) or all-cause mortality. Fifty-eight patients (12.7%) died and 49 (10.7%) experienced MACEs during follow-up. The most notable finding was that progression of hyaline arteriolar thickening (aah) between 3 and 12 months independently predicted all-cause mortality, even after adjustment for estimated glomerular filtration rate, diabetes, and previous cardiovascular disease. In addition, vascular fibrous intimal thickening at 12 months was also independently associated with mortality, while associations of baseline vascular or interstitial lesions were attenuated after full multivariable adjustment. These results suggest that progressive aah reflects an ongoing recipient-related vascular process rather than donor-derived injury. Monitoring this dynamic histological change in repeated biopsies performed for protocol or for cause may provide transplant nephrologists with an early signal of increased mortality risk.

## 1. Introduction

Kidney transplantation stands out as the optimal therapeutic choice for end-stage kidney disease (ESKD) patients, offering superior outcomes in terms of both mortality and quality of life. Despite these advantages, recipients of kidney transplants continue to face elevated rates of cardiovascular events [1], constituting the primary cause of mortality among this group [2]. The heightened incidence of cardiovascular outcomes in ESKD is attributed to a multitude of factors, including diminished renal function and prevalent comorbidities such as hypertension, diabetes mellitus, heart failure, vascular calcification, and atherosclerosis [3,4].

Assessing kidney graft biopsies is crucial for interpreting information within the context of renal function [5]. Protocol biopsies are primarily performed in many centers to detect subclinical rejection. However, they may also provide valuable information on chronic vascular and interstitial remodeling that reflects systemic processes beyond the allograft itself. Evidence suggests that urinary fibrosis markers and vascular calcification are independently associated with an increased risk of cardiovascular events and death, irrespective of eGFR [6,7]. Whether chronic histological changes in protocol allograft biopsies provide prognostic information for patient outcomes beyond graft survival remains uncertain.

This study aimed to address this gap by assessing the impact of vascular and interstitial changes on long-term mortality in kidney transplant recipients (KTRs).

## 2. Materials and Methods

### 2.1. Study Design and Participants

Every KTR from Hospital Clínic of Barcelona who received a kidney transplant between March 2015 and March 2019 was considered for inclusion in this prospective observational study. Seventy-two patients were not analyzed either because they got lost to follow-up during the study time or due to early graft loss, defined as graft failure within the first 30 days after kidney transplantation. The subjects included were followed from transplantation day until March 2023.

### 2.2. Variables

The variables were collected from electronic health records. They consisted of demographic (age, gender, and ethnicity), past renal history (time on kidney replacement therapy, baseline etiology of kidney disease, transplant number, and type of donor), comorbidities at the time of transplantation (hypertension, diabetes mellitus, dyslipidemia, body mass index, heart failure, coronary artery disease, and smoking status), laboratory tests (creatinine, phosphate, 25-hydroxy vitamin D, and parathyroid hormone [PTH]), induction and maintenance immunosuppressive therapy, and protocol allograft biopsy findings at 3 and 12 months after transplantation. Hypophosphatemia and hyperphosphatemia were defined as ≤2.5 mg/dL and ≥4.5 mg/dL, respectively. eGFR was calculated with the CKD Epidemiology Collaboration (CKD-EPI) formula [8], and pathology findings were reported according to the Banff classification [9]. The individual scores for interstitial fibrosis and tubular atrophy were added to determine IFTA, the scores for glomerulitis and mesangial expansion to determine glomerulopathy and arteriolar hyalinosis with vascular fibrous intimal thickening to determine vasculopathy [6]. Creatinine and phosphate levels were registered at the three time points (baseline or day of transplantation, three months after transplantation coinciding with the first protocol graft biopsy, and 12 months after transplantation coinciding with the second protocol biopsy). 25-hydroxy vitamin D and PTH were registered at baseline and 12 months after transplantation. Progression of histopathological lesions was evaluated only in patients with paired 3- and 12-month protocol biopsies. Patients with only one available biopsy were excluded from progression analyses. Two experienced renal pathologists independently scored the biopsies according to Banff 2018 [9], with discrepancies resolved by consensus.

### 2.3. Study Outcomes

All-cause mortality and the composite outcome of major adverse cardiovascular events (MACEs) that included myocardial infarction, acute coronary syndrome, stroke, hospitalizations for heart failure, and all-cause and cardiovascular death that occurred during follow-up were recorded. Pathology alterations were reported according to the Banff Classification of Allograft Pathology [9,10].

### 2.4. Statistical Analysis

Quantitative data are presented as mean and standard deviation when normally distributed, and as median and interquartile range otherwise. Qualitative variables are represented as absolute and relative frequencies. The statistical inference between groups was performed by Student’s *t*-test when quantitative variables were normally distributed, while the Mann–Whitney test was used for skewed data. Differences in qualitative variables were analyzed with the χ^2^ test or Fisher’s exact test when one or more expected values were less than five or the data were very unequally distributed among the table’s cells. Cox proportional hazards regression was used to explore the relationship between the survival event-free time of patients and one or more predictor variables. Statistical significance was assumed when the *p*-value was inferior to 0.05 on all statistical tests. Statistical analysis was performed with IBM’s SPSS Statistics software in its 26th version.

### 2.5. Institutional Review Board Statement and Ethical Statement

This study has been evaluated and approved by the Clinical Research Ethics Committee of the Hospital Clínic of Barcelona (HCB/2020/0380, 30 April 2020). The data collection has followed the Regulation (EU) 2016/679 (General Data Protection Regulation), its subordinate national and regional laws, the Declaration of Helsinki principles, and the principles of the Declaration of Istanbul as outlined in the ‘Declaration of Istanbul on Organ Trafficking and Transplant Tourism.’

## 3. Results

### 3.1. Baseline Characteristics

**Figure 1:**
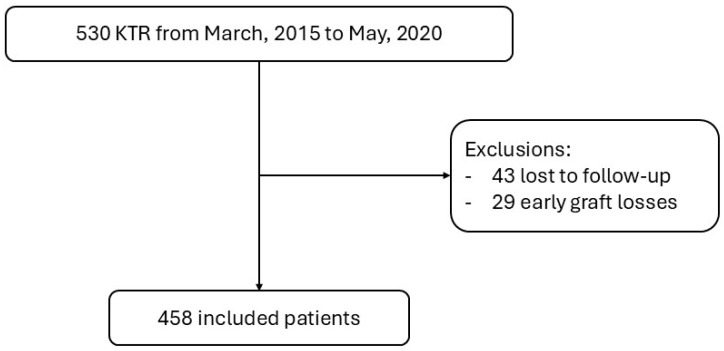
Flow diagram with the number of individuals and reasons for exclusion or loss to follow-up.

A total population of 530 patients received a kidney transplant between March 2015 and May 2020. Seventy-two patients were excluded from the analysis because of the causes detailed in Figure 1. Among the 458 patients included, 159 had paired 3- and 12-month protocol biopsies, which were used for progression analyses. Ethnicity, age, comorbidities, type of donor, and other included population characteristics are further detailed in Table 1.

### 3.2. Vascular Lesions

One hundred and ninety-eight (68.9%), 24 (6.2%), and 79 (27%) patients had vascular fibrous intimal thickening (cv), hyaline arteriolar thickening (aah), and arteriolar hyalinosis (ah) scores greater than zero, respectively. By the twelve-month biopsy, these vascular alterations had progressed in 29.2%, 9.4%, and 23.3% of patients, respectively. Patients with a history of hypertension had worse cv scores at the three-month biopsy (71.7% vs. 56%, *p* = 0.026). Recipients of deceased-donor kidneys showed higher cv scores at three months (75.8% vs. 53.4%, *p* < 0.001) and higher cv and ah scores at twelve months (74.9% vs. 56.1%, *p* = 0.011; and 40% vs. 22.9%, *p* = 0.005, respectively) compared with those receiving living-donor kidneys. Patients with diabetes or hyperphosphatemia had higher aah scores at three months (11.2% vs. 7.2%, *p* = 0.009; and 16.7% vs. 7.7%, *p* = 0.035, respectively). Those with dyslipidemia had higher ah scores at twelve months (40% vs. 26.2%, *p* = 0.039). No significant differences in vasculopathy were observed between smokers and non-smokers; however, active smokers more frequently showed progression of aah from three to twelve months (30.8% vs. 7.5%, OR 5.45 [1.45–20.59], *p* = 0.006).

### 3.3. Interstitial Fibrosis and Tubular Atrophy (IFTA)

At three months post-transplantation, 206 patients (44.6%) had tubular atrophy (ct) and 148 (32.3%) had interstitial fibrosis (ci) greater than zero. Only 29% and 14% of patients showed neither lesion at the three- and twelve-month biopsies, respectively. By twelve months, IFTA had progressed in 69 patients (15.1%) with ci and in 58 patients (12.7%) with ct. Progressive ci was significantly associated with maintenance immunosuppressive therapy including mTOR inhibitors (43.6% vs. 56.4%, *p* = 0.042), and with a lower eGFR at twelve months (44.8 vs. 52.6 mL/min/1.73 m^2^, *p* = 0.004).

### 3.4. All-Cause Mortality

**Figure 2:**
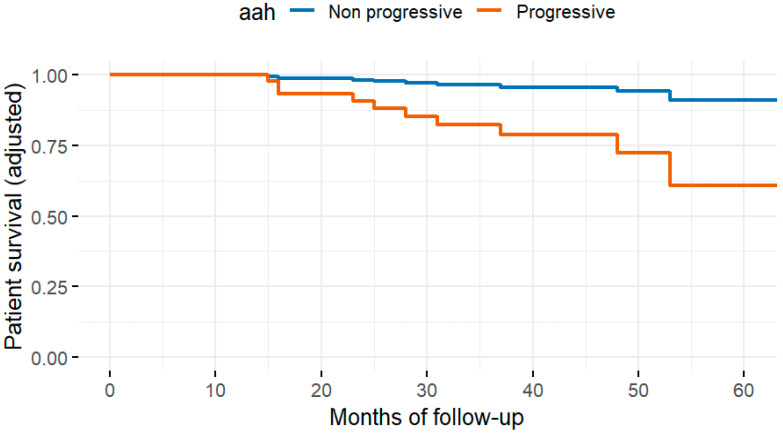
Adjusted patient survival curves according to the presence of arteriolar hyalinosis (aah), derived from the multivariable Cox model. The analysis was adjusted for age, diabetes, cardiovascular history, dialysis vintage, and estimated glomerular filtration rate at baseline and 12 months. Patients with aah (red line) showed significantly lower adjusted survival probabilities (*p* = 0.017).

**Figure 3:**
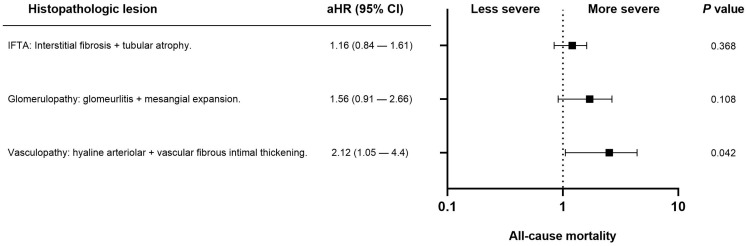
Forest plot showing the association between chronic histopathologic lesions and all-cause mortality in kidney transplant recipients. Adjusted hazard ratios (aHR) with 95% confidence intervals (CI) are derived from multivariable Cox regression models adjusted for estimated glomerular filtration rate, diabetes, cardiovascular history, age, and dialysis vintage. Only vasculopathy (arteriolar hyalinosis + vascular fibrous intimal thickening) showed a statistically significant association with mortality (*p* = 0.042).

Fifty-eight patients (12.7%) died during follow-up. The most common cause of death was infection (22, 4.8%), followed by cancer (11, 2.4%), cardiovascular disease (10, 2.2%), and other causes (15, 3.3%). In univariate analysis, the histopathologic variables associated with all-cause mortality were interstitial fibrosis at three months (ci > 0), progressive hyaline arteriolar thickening (aah) between three and twelve months, and vascular fibrous intimal thickening at twelve months (cv > 1). After multivariable adjustment in the four Cox regression models, progressive aah at twelve months and 12-month (Figure 2) cv > 1 remained significantly associated with mortality (Table 2). When vascular, glomerular, and tubulointerstitial lesions were analyzed as grouped variables without applying a cut-off, vasculopathy showed a significant association with all-cause mortality after adjustment for eGFR, diabetes, cardiovascular history, age, and dialysis vintage (Figure 3).

### 3.5. Cardiovascular Events

Fatal cardiovascular events occurred in 10 patients (2.2%), non-fatal events in 44 (9.6%), and MACEs in 49 (10.7%). A three-month cv score greater than 2 was significantly associated with the occurrence of fatal and non-fatal events as well as MACEs. This association remained significant after adjustment for eGFR, diabetes, and previous cardiovascular history, but was no longer significant after adjustment for age and dialysis vintage (Table 2).

### 3.6. Other Clinical Variables

To assess the independent associations between histopathologic findings and clinical outcomes, we constructed multivariable Cox regression models. Covariates were selected based on their clinical relevance and statistical significance in univariate analyses. The final models included variables that remained significant in the multivariate analysis, as shown in Table 3 and Table 4. A history of cardiovascular disease [HR 4.3, 95% CI (2.45–7.53), *p* <0.001], *p* = 0.01], recipient age [HR 1.05, 95% CI (1.03–1.08), *p* < 0.001], hypophosphatemia [HR 2.5, 95% CI (1.25–5.05), *p* = 0.01], and dialysis vintage [HR 1.003, 95% CI (1–1.005), *p* = 0.003] were associated with an increased risk of MACEs, while history of cardiovascular disease [HR 3.84, 95% CI (2.08–7.09), *p* < 0.001], and recipient age [HR 1.05, 95% CI (1.02–1.08) *p* = 0.001) were associated with a non-fatal cardiovascular event.

## 4. Discussion

In this study, we found that progression of aah between 3 and 12 months was independently associated with all-cause mortality, highlighting the role of ongoing recipient-related vascular remodeling. Vascular fibrous intimal thickening at 12 months also predicted mortality. Furthermore, recipients with smoking, dyslipidemia, diabetes, hyperphosphatemia, or hypertension, as well as those who received a deceased-donor graft, were more likely to develop chronic vascular lesions

Our cohort’s overall incidence of cardiovascular events was similar to those previously reported in developed countries, as we found 10.7% of MACEs, while others reported 8.3% [11]. However, the most frequent cause of death in our cohort was infections, which, in fact, doubled the number of cases of cardiovascular death and cancer, respectively. A higher number of deaths related to infection was reported in other cohorts, although the difference was still smaller than in our population compared to cardiovascular disease or malignancy [12,13]. Infection-related deaths may partly reflect the inclusion of follow-up years coinciding with the COVID-19 pandemic, which substantially affected immunosuppressed populations [14].

In our study, the most relevant finding was that progression of aah between 3 and 12 months independently predicted all-cause mortality. Both ah and aah are scored within the Banff classification, though they capture related but distinct patterns of vascular injury. While ah reflects hyaline deposition within the arteriolar wall and may result from multiple factors such as hypertension, diabetes, or calcineurin inhibitor exposure, aah describes a concentric thickening of the arteriolar media. Importantly, aah was introduced in the Banff classification to improve reproducibility and diagnostic value in the assessment of chronic vascular lesions. The progression of this lesion is unlikely to represent donor-derived injury and instead suggests an ongoing recipient-related vascular process. Vascular fibrous intimal thickening at 12 months was also independently associated with mortality, although changes between month 3 and 12 of this parameter were not correlated with adverse outcomes. An inflammatory environment that could induce systemic vascular remodeling and endothelial dysfunction with poor long-term outcomes in transplant recipients [15]. Our findings extend previous observations in non-transplant CKD cohorts, where arteriolar or arterial sclerosis predicted cardiovascular events and death [6]. Unlike this study, we demonstrate that, in KTRs, the progression of hyaline arteriolar thickening, rather than baseline lesions, provides independent prognostic information for patient survival.

There are multiple hits that could lead to graft vasculopathy. For instance, there is evidence that associates late rejection in living KTRs with cardiovascular events, though they do not specify on the histopathologic lesions found [16]. Also, inflammation coming from the donated kidney and aggravated by ischemia–reperfusion injury may induce a vicious cycle of fibrosis and inflammation where fibroblasts release pro-inflammatory cytokines (e.g., TNF-α, IL-1, IL-6) and growth factors (like TGF-β) [17,18], which may further stimulate inflammation elsewhere. In our cohort specific cytokine or fibrosis biomarkers were not available, as they are not routinely measured during standard clinical follow-up. Future studies including stored samples could further clarify these associations. Additionally, immunosuppressive treatment with calcineurin inhibitors induces oxidative stress and impairs vasodilation by decreasing fibrinolytic activity in vessel walls, increasing intracellular calcium in vascular smooth muscle cells, contributing to vascular stiffness, inflammation, and fibrosis [19,20,21]. These changes in vascular remodeling likely accelerate vasculopathy and compromise graft and patient survival [19,22].

The role of inflammation, particularly vascular inflammation and cardiovascular disease, is well established. Matrix metalloproteinases and cytokines, which are upregulated with aging and in response to chronic immunosuppression, promote vascular remodeling and intimal thickening [23,24,25,26,27]. This remodeling can lead to narrowing of the vessel lumen, reducing end-organ perfusion, and potentially exacerbating cardiovascular risk [28,29]. We did not find an association between maintenance immunosuppressive treatment and vasculopathy or mortality and MACEs. This supports current knowledge despite preclinical studies demonstrated that sirolimus attenuated vascular and myocardial fibrosis [30,31], and everolimus improved post-myocardial infarction remodeling [30,32]. The impact of mTOR inhibitors on hard cardiovascular outcomes has been mixed [33,34,35], though many studies were underpowered to detect differences in cardiovascular outcomes [28].

To our knowledge, this is the first work that has looked for a relationship between cardiovascular events and all-cause mortality with histopathologic findings on protocol kidney graft biopsies. Nevertheless, there is a recent publication where the authors looked for a similar association, although it was held in CKD patients, not kidney recipients [6]. There, they found a significant association between mesangial expansion, IFTA, and arterial and arteriolar sclerosis with MACEs and death. Although when adjusting for other factors, the only variables that remained significant were mesangial expansion and arterial sclerosis [6]. We did not find any significant association between mesangial expansion and mortality or MACEs. As populations were different, where one had a longer CKD history of disease and was mostly not under immunosuppressants, these findings are not fully comparable. Still, they show a relationship between histology findings and systemic disease manifestations and vice versa [23,36].

Concerning other related factors, we found that hyperphosphatemia, active smoking, and diabetes were associated with vascular lesions, as has been described in non-transplanted CKD patients [37,38,39,40]. However, these factors alone were not independently associated with MACEs, or patient survival during our study’s follow-up. Curiously, we found that pre-transplantation hypophosphatemia was significantly associated with MACEs, something that has been described in the general population [41,42] but not in KTRs [43]. We believe that these findings warrant further research as they may be associated with malnutrition and other potentially related causes (e.g., repeated admissions, chronic inflammation) that fall beyond the scope of this paper.

This study has several limitations. It was conducted at a single center with a moderate sample size, and not all patients underwent both protocol biopsies. Protocol biopsies also provide information at specific time points and may not fully capture dynamic changes. Renal function may have been overestimated, as transplant recipients often lose muscle mass due to chronic steroid use or comorbidities, and cystatin C–based eGFR was not available [44,45]. Donor-related risk factors were not collected, and information on concomitant therapies after biopsy findings (e.g., statins, RAAS inhibitors, aspirin) was lacking. Follow-up time, although up to eight years, may still have been insufficient for some events to occur, and given that many patients were transplanted outside our autonomous region, some events may not have been recorded. Despite these limitations, the use of standardized Banff scoring, prospective data collection, and robust multivariable models strengthen the validity of our results.

## 5. Conclusions

In conclusion, protocol kidney allograft biopsies provide prognostic information that extends beyond rejection monitoring. Among chronic histological lesions, progression of hyaline arteriolar thickening between 3 and 12 months emerged as an independent predictor of all-cause mortality, supporting the biological plausibility of an active, recipient-related vascular process rather than donor-derived damage. Vascular fibrous intimal thickening at 12 months was also associated with mortality, but only aah progression offered a dynamic signal of ongoing vascular remodeling. These findings suggest that repeated biopsy assessment may not only inform graft status but also serve as a window into systemic cardiovascular vulnerability in KTRs. Until further studies are performed, the management of cardiovascular risk factors should be optimized in these individuals. Larger studies are needed to validate these observations and explore how histological monitoring might be integrated into cardiovascular risk stratification after transplantation.

## Figures and Tables

**Table 1:** Demographic and clinical baseline characteristics of study participants.

Variable	Study Population (*n* = 458)
Age, median (IQR)	58 (48.8–67)
Male sex, n (%)	281 (61.4%)
Race, n (%)	
White	435 (95%)
Latin American	16 (3.5%)
Asian	5 (1.1%)
Black	2 (0.4%)
Smoking, n (%)	46 (10%)
Pre-transplant diabetes, n (%)	
Type 1	24 (5.2%)
Type 2	100 (21.8%)
Pre-transplant hypertension, n (%)	386 (84.3%)
Pre-transplant BMI, Kg/m^2^, median (IQR)	25.3 (6.93)
Pre-transplant cardiovascular disease, n (%)	81 (17.7%)
Pre-transplant hypophosphatemia, n (%)	43 (9.5%)
Pre-transplant hyperphosphatemia, n (%)	225 (49.5%)
Pre-transplant vitamin D deficiency, n (%)	200 (44.4%)
Pre-transplant dyslipidemia, n (%)	196 (42.8%)
Deceased donor, n (%)	
DCD	150 (32.7%)
DBD	173 (37.8%)
Dialysis vintage, months, median (IQR)	30 (9.8–103.3)

DBD, dead brain donor; DCD, donor after circulatory death; IQR, interquartile range.

**Table 2:** Association of chronic histopathologic lesions with all-cause mortality and fatal and non-fatal cardiovascular outcomes.

Variable	Overall n (%)	Unadjusted		Adjusted—Model 1 (eGFR)		Adjusted—Model 2 (DM and CV History)		Adjusted—Model 3 (Age and Dialysis Vintage)		Adjusted—Model 4 (Models 1–3)	
		HR (CI 95%)	*p*-Value	HR (CI 95%)	*p*-Value	HR (CI 95%)	*p*-Value	HR (CI 95%)	*p*-Value	HR (CI 95%)	*p*-Value
All-cause mortality	58 (12.7%)										
3-m. Interstitial fibrosis > 5%	148 (32.3%)	2.5 (1.05–5.06)	0.038	2.57 (0.88–7.51)	0.084	2.47 (1.02–5.98)	0.045	2.32 (0.96–5.6)	0.06	2.34 (0.89–6.14)	0.084
Progressive hyaline arteriolar thickening	15 (9.4%)	4.7 (1.21–18.32)	0.026	5.03 (1.23–19.64)	0.02	4.65 (1.17–18.47)	0.029	5.65 (1.4–22.8)	0.015	5.6 (1.36–23.19)	0.017
12-m. Vascular fibrous intimal thickening > 25%	45 (18.4%)	3.13 (1.23–7.56)	0.01	2.94 (1.14–7.56)	0.026	3.78 (1.49–9.6)	0.005	2.62 (1.08–6.38)	0.034	3.33 (1.27–8.7)	0.015
Non-fatal cardiovascular events	44 (9.6%)										
3-m. Vascular fibrous intimal thickening > 50%	18 (6.3%)	5.98 (1.34–26.66)	0.019	5.31 (1.13–24.92)	0.034	9.65 (1.98–47.06)	0.005	3.03 (0.62–14.74)	0.17	5.17 (0.86–31.26)	0.073
Cardiovascular death	10 (2.2%)										
3-m. Vascular fibrous intimal thickening > 50%	3 (1%)	18.23 (1.64–203.26)	0.018	24.61 (1.44–420)	0.027	14.47 (1.31–160.4)	0.029	20.44 (0.47–886)	0.117	21.38 (0.35–1308)	0.145
MACE	49 (10.7%)										
3-m. Vascular fibrous intimal thickening > 50%	19 (6.6%)	5.09 (1.16–22.39)	0.031	5.05 (1.08–23.65)	0.04	8.64 (1.8–41.34)	0.007	2.21 (0.45–10.79)	0.33	3.84 (0.62–23.95)	0.15

3-m., 3-month; 12-m., 12-month; CI, confidence interval; CV, cardiovascular; DM, diabetes mellitus; eGFR, estimated glomerular filtration rate; HR, hazard ratio; MACE, major adverse cardiovascular event.

**Table 3:** Association of all-cause mortality with patients’ demographic and clinical characteristics during follow-up.

Variable	Dead n = 58 (12.7%)	Living n = 400 (87.3%)	Univariate Analysis		Multivariable Analysis	
			*p*-Value	Odds Ratio or Mean/Median Difference (CI 95%)	*p*-Value	Exponentiated ß-Coefficient (CI 95%)
Age, median (IQR)	68 (61.5–70.3)	56 (47–65)	<0.001	10.47 (13.93–7.02)	<0.001	1.08 (1.05–1.11)
Male sex, n (%)	37 (63.7%)	244 (61%)	0.683	0.89 (0.5–1.57)		
Smoking, n (%)	9 (15.5%)	37 (9.3%)	0.138	1.8 (0.82–3.96)		
Dyslipidemia, n (%)	31 (53.4%)	165 (41.3%)	0.079	1.64 (0.94–2.84)		
Deceased donor, n (%)	49 (84.5%)	274 (68.5%)	0.013	2.26 (1.14–4.47)	0.9	0.99 (0.79–1.22)
3-month eGFR, median (IQR)	39 (25.8–55.3)	47 (33–64)	0.022	3.45 (−2.71–9.61)	0.036	0.99 (0.97–0.99)
12-month eGFR, median (IQR)	41 (31.5–55.5)	49 (36–65)	0.041	5 (−0.73–10.73)	0.06	0.98 (0.97–1)
Dialysis vintage, months, median (IQR)	34 (14.8–66.5)	29 (98.3)	0.807	3.87 (−27.19–34.94)	0.72	1 (0.99–1)
Pre-transplant hypertension, n (%)	54 (93.1%)	332 (83%)	0.048	2.77 (0.97–7.9)		
Pre-transplant BMI, Kg/m^2^, mean ± SD	25.47 ± 4.5	25.91 ± 5.36	0.55	0.44 (−1.02–1.91)		
Pre-transplant diabetes, n (%)	26 (44.8%)	98 (24.5%)	0.001	2.5 (1.42–4.41)	0.002	2.31 (1.37–3.9)
Pre-transplant cardiovascular disease, n (%)	20 (34.5%)	61 (15.3%)	< 0.001	2.93 (1.6–5.37)	0.01	2.06 (1.19–3.55)
Pre-transplant hypophosphatemia, n (%)	10 (17.2%)	33 (8.3%)	0.006	2.9 (1.33–6.33)	0.46	1.35 (0.61–2.99)

SD, standard deviation; BMI, body mass index; CI: confidence interval; IQR, interquartile range.

**Table 4:** Association of major adverse cardiovascular outcomes with patients’ demographic and clinical characteristics during follow-up.

Variable	MACEs n = 49 (10.7%)	No MACEs n = 409 (89.3%)	Univariate Analysis		Multivariable Analysis	
			*p*-Value	Odds Ratio or Mean/Median Difference (CI 95%)	*p*-Value	Exponentiated ß-Coefficient (CI 95%)
Age, median (IQR)	65 (58–69.5)	56 (48–66)	<0.001	−7.56 (−11.35–−3.76)	<0.001	1.05 (1.03–1.08)
Male sex, n (%)	35 (71.4%)	246 (60.1%)	0.13	0.6 (0.32–1.16)		
Smoking, n (%)	3 (6.2%)	43 (10.5%)	0.33	0.56 (0.17–1.86)		
Dyslipidemia, n (%)	25 (51%)	171 (41.8%)	0.22	1.45 (0.80–2.63)		
Deceased donor, n (%)	40 (81.6%)	283 (69.2%)	0.07	1.84 (0.92–3.69)		
DBD, n (%)	25 (51%)	148 (36.2%)				
DCD, n (%)	15 (30.6%)	135 (33%)				
12-month PTH, median (IQR)	321.45 ± 297.52	224.98 ± 232.75	0.05	−96.48 (−173.39–−19.57)	0.016	1 (1–1.002)
3-month eGFR, median (IQR)	43.5 (30–59)	47 (32–63.5)	0.27	3.45 (−2.71–9.61)		
12-month eGFR, median (IQR)	44 (35.3–54.8)	49 (36–66)	0.09	5 (−0.73–10.73)		
Dialysis vintage, months, median (IQR)	57 (31–191.5)	27 (7.5–84)	0.02	−53.58 (−86.64–−20.51)	0.003	1 (1–1.01)
Pre-transplant hypertension, n (%)	47 (95.9%)	2 (0.4%)	0.02	4.85 (1.15–20.45)	0.051	4.08 (0.99–16.8)
Pre-transplant BMI, Kg/m^2^, mean ± SD	26.04 ± 4.66	25.84 ± 5.33	0.8	−0.2 (−1.79–1.39)		
Pre-transplant diabetes, n (%)	24 (48.9%)	25 (6.1%)	< 0.001	2.97 (1.62–5.43)	<0.001	2.89 (1.62–4.98)
Pre-transplant cardiovascular disease, n (%)	25 (51%)	56 (13.7%)	< 0.001	6.57 (3.51–12.29)	<0.001	4.3 (2.45–7.53)
Pre-transplant hypophosphatemia, n (%)	10 (20.4%)	33 (8.1%)	0.006	2.9 (1.33–6.33)	0.01	2.5 (1.25–5.05)

BMI, body mass index; CI: confidence interval; eGFR, estimated glomerular filtration rate; SD, standard deviation.

## Data Availability

The data supporting the findings of this study are available on GitHub (https://github.com/Broseta/Kidney-trasnplantation/blob/main/datos.csv).

## Abbreviations

The following abbreviations are used in this manuscript:

aah	Hyaline Arteriolar Thickening
ah	Arteriolar Hyalinosis
CKD	Chronic Kidney Disease
ci	Interstitial Fibrosis
CKD-EPI	Chronic Kidney Disease Epidemiology Collaboration
ct	Tubular Atrophy
cv	Vascular Fibrous Intimal Thickening
eGFR	Estimated Glomerular Filtration Rate
ESKD	End-Stage Kidney Disease
IFTA	Interstitial Fibrosis and Tubular Atrophy
KTR	Kidney Transplant Recipient
MACEs	Major Adverse Cardiovascular Events
PTH	Parathyroid hormone
